# Exploring factors affecting the use of blended diets for children and young people in health, education and social care settings in the UK: The protocol for a realist mixed methods study

**DOI:** 10.1177/02601060261439096

**Published:** 2026-04-08

**Authors:** Gemma Phillips, Jane Coad, Mary Hickson, Joseph C Manning

**Affiliations:** 1 School of Healthcare, 4488 University of Leicester, Leicester, UK; 2 Leicestershire Partnership NHS Trust, Leicester, UK; 3 6123 University of Nottingham, Nottingham, UK; 4 62641 University of Plymouth, Plymouth, UK; 5 Nottingham Children's Hospital, 9820 Nottingham University Hospitals NHS Trust, Nottingham, UK

**Keywords:** Blended diet, children, young people and families, enteral tube feeding, mixed methods research, realist enquiry

## Abstract

**Background:**

Enteral tube feeding provides supplementary or alternative nutrition for individuals who cannot meet their nutritional needs orally and remains essential for a number of children and young people with complex health needs. Commercial formula remains standard practice in the UK, although increasing numbers of children and their families are choosing a blended diet and reporting significant physical and social benefits. However, support for families in using blended diets varies across health, education and social settings in the UK.

**Aim:**

To explore factors affecting the use of blended diets in health, education and social care settings.

**Methods:**

This sequential mixed methods study uses a realist approach to understand what works for who under what circumstances and why. A point prevalence study of blended diet use in the UK will identify a stratified sample of professionals and families for interviews exploring their experiences. A Delphi study will prioritise key issues identified in the interview data.

**Summary:**

This paper presents a novel, realist mixed methods protocol to investigate the contextual factors influencing the use of blended diets for children and young people across health, education and social care settings in the UK. Grounded in lived-experience, it addresses a critical gap in the evidence-base responding to family-identified priorities. Integrating qualitative and quantitative data enhances explanatory power and applicability to practice. Findings will inform future interventions and policy development, aiming to improve support and equity in blended diet provision. This research offers a robust foundation for advancing personalised enteral feeding approaches within multidisciplinary care.

## Plain English summary

A number of children and young people with complex health needs require tube feeding. Commercial formula is the standard option in the UK. Although, some families prefer blended real food, because it can reduce symptoms such as vomiting and help children feel more included during mealtimes. However, places like schools and hospitals vary in the support they provide for blended diets. This creates challenges where staff may be unfamiliar with or restricted from using blended diets. The study aims to explore what helps or hinders the use of blended diets in different settings. The research will be divided into three parts:

1. Information about how many people use a blended diet will be collected from the NHS.
2. Structured conversations with people who have experience of tube feeding. These people can be parents or main carers, young people or professionals who work with them.
3. Asking a group of experts* to identify and prioritise the most important issues raised in the interviews.

The asterisk indicates that the experts will be parents or main carers, young people or professionals who work with them. This research is novel in that it will help to understand how blended diets are supported outside the home. This can inform future guidelines and policies so that people can be helped to use a blended diet in places like schools and hospitals. This study shows how mixed methods can be used to do research that can inform healthcare guidelines and policy.

## Background

Enteral tube feeding is a method of providing nutrition and fluids for children and young people who are unable to take enough food and drinks by mouth and are therefore at risk of malnutrition (NICE, 2006). Underlying conditions such as cerebral palsy, which accounts for a significant proportion of gastrostomy placements in the UK, often result in the inability to manage sufficient oral intake (Popescu and Mutalib, 2023). Enteral tube feeding can be used as a sole source of nutrition for those unable to swallow safely or in addition to an oral diet where supplementary nutrition is necessary.

Enteral tube feeding dates back centuries; however, technological advancements in the 1950s have shaped current enteral tube feeding practices (Harkness, 2002). During this period, enteral tube feeding was used most during periods of acute illness in hospital settings. More recently, the use of home enteral tube feeding has increased significantly as children and young people are living longer with complex medical needs necessitating long term enteral tube feeding outside of a hospital environment. Many of these individuals have life-long conditions, in contrast to acute illnesses, and require support from carers and other health and care services. Although the feeding methods itself does not differ, community settings present distinct priorities and challenges.

Guidelines in the UK advise using a commercial formula for enteral tube feeding (NICE, 2006). Commercial formulas are ready-made, liquid formulas available on prescription in the UK. They are nutritionally complete in a specified volume and can be given as a bolus or using a pump. However, increasingly individuals and their families are choosing to use blended diets and reporting an array of benefits associated with this practice (Breaks et al., 2018; McCormack et al., 2023). A blended diet consists of everyday foods blended to a puree in addition or as an alternative to commercial formula (Durnan et al., 2021). Blended diets are most commonly used with gastrostomy tubes which are therefore the focus in this study; current guidance cautions against using them with nasogastric and post-pyloric tubes (Durnan et al., 2021). Families report benefits such as reduced gastrointestinal symptoms and greater inclusion in family mealtimes. In addition, families of children with complex needs highly value the normalisation of gastrostomy feeding (Durnan, 2018; Phillips and Coad, 2023).

Families often choose blended diets because commercial formula is poorly tolerated which highlights the need for alternatives (Phillips and Coad, 2023). Families report a ‘radical change’ after commencing a blended diet although a lack of support to use them (Durnan, 2018). This ‘radical change’ was associated with reduced reflux and vomiting, healthy growth and improved wellbeing. Some studies have associated the use of a blended diet with improvement (95%) in gastrointestinal symptoms (Batsis et al., 2020) whilst others have demonstrated a reduction (39%) in those using medications to manage these symptoms following transition to a blended diet (Kernizan et al., 2020). Although the mechanisms remain unclear, some authors hypothesise that changes in the gut microbiome as a result of a blended diet may contribute to these changes (Marchesi et al., 2022). This is pertinent to children and young people whose gut flora may lack diversity due to extensive enteral tube feeding from an early age (Marchesi et al., 2022). These changes to the gut microbiome may also improve general health and wellbeing (Gallagher et al., 2018). In addition, blended diets have also been shown to reduce the frequency of respiratory infections and access to acute healthcare facilities which are common amongst individuals with neurodisability (Hron et al., 2019).

Furthermore, the impact of using blended diets on the wider family can be significant. Parents report feeling empowered, and value the normalisation of gastrostomy tube feeding associated with using a blended diet (Soscia et al., 2021). Normalisation is particularly important given the complexity of the needs of many of these individuals and the duration of enteral tube feeding which for some may be life-long. Non-oral feeding has been shown to have significant impact on relationships between mothers and their children (Wilken, 2012). This sense of loss associated with enteral tube feeding is a key factor influencing parents/carers’ decision to use a blended diet for their child (Breaks et al., 2022).

Nutritional inadequacy and tube blockage are common concerns related to using blended diets. However, studies show that adverse events happen less frequently and are less problematic than anticipated (Armstrong et al., 2017). The protein and fat content of blends have been shown to be comparable to commercial formula with blended diets offering greater fibre content (Gallagher et al., 2018; Ojo et al., 2020). Nutritional deficiencies can occur with both commercial formula and blended diets with professional oversight shown to mitigate this risk (Breaks et al., 2018; Schultz and Kim, 2024). In addition, parents/carers report greater satisfaction with support from professionals as opposed to social groups (Staunton et al., 2023).

Current British Dietetic Association guidance now encourages open discussions with families and the multidisciplinary team about blended diets to support informed decision making (Durnan et al., 2021). Previously, blended diets were actively advised against, and early re-emergence was met with caution. Despite updated guidance, families continue to encounter barriers when using blended diets (Armstrong et al., 2017). Studies show that resistance from health and social care professionals towards blended diets remains (Phillips et al., 2024). Whilst guidance suggests that dietitians should lead multi-professional discussions around blended diets, only 35% (n = 51) of dietitians in a study by O'Connor et al. (2025) reported feeling confident or very confident to review a child using a blended diet. Certain organisations continue to prohibit blended diets and families feel frustrated by this lack of support with many ‘forced to compromise’ (Breaks et al., 2018). Anecdotal evidence suggests that support for blended diets varies across the UK, resulting in inequitable service provision.

There is little published research relating to the use of blended diets being used in health, education and social care settings. The increased use in the UK has been driven by families as opposed to being introduced in the same way as other healthcare interventions through design, development and rigorous testing using randomised trials. The majority of the current evidence is observational which limits certainty of causation. However, choices about enteral tube feeding for children and young people, many with additional needs, are complex. Consequently, it is unlikely that a single method of feeding is superior which challenges the concept of a ‘gold standard’. Therefore, options are necessary so that feeding practices can be personalised to individual children, young people and their families and they are supported to make informed choices. Research on blended diets must therefore consider this complexity.

Patients and families using blended diets also report a range of benefits including symptom relief and social inclusion. The increase in the use of blended diets is largely driven by families seeking an alternative to commercial formula for their tube-fed child. These families continue to receive insufficient support across these settings which directly informed the development of this study.

In summary, blended diets are becoming increasingly common and they have the potential to improve health and quality of life for patients and their families. Research has demonstrated positive patient, carer and family experiences in the absence of consistent support which can be problematic. This study builds on other recent work in the field exploring health outcomes, resource implications, benefits and complications of blended diets for gastrostomy feeding (Fraser et al., 2024; McCormack et al., 2023).

The proposed study focuses on the implementation of blended diets and comprises the early stages of complex intervention development to optimise the support families receive to use blended diets. Complex interventions are required for complex health and social problems and often have multiple interacting components which operate in dynamic situations. Complex intervention development consists of four stages: identification, feasibility, evaluation and implementation (Skivington et al., 2021). The identification stage focuses on understanding as a foundation for subsequent development. An understanding the wider context is key to developing effective interventions in health and social care and applies to all disciplines. This study is distinctive in its approach to encompass health, education and social care services aligning to government strategy to embed integrated care systems and coordinate services (Bliss et al., 2024). This is pertinent to children and young people with complex care needs as it is likely that they access a multitude of services across health and social care. Families report that segregation between services, health and social care can hinder holistic care for their child or young person impacting the whole family.

## Aim

To explore factors affecting the use of blended diets for children and young people in health, education and social care settings in the UK.

Objectives:

1. Identify the prevalence of the use of blended diets for children and young people across the UK.
2. Explore views and experiences of health, education and social care professionals and families to determine the context and underlying mechanisms affecting the use of blended diets.
3. Identify and prioritise components of an intervention to support the delivery of blended diets in health, education and social care settings.

## Methods

This sequential mixed methods study follows a realist evaluation approach outlined by Pawson and Tilley (1997). It is a theory-driven approach which moves beyond evaluating effectiveness to explore how contextual factors trigger mechanisms to generate outcomes. The approach is grounded in critical realism which assumes a stratified reality influenced by social structures (Bhaskar, 1975). Critical realism accepts the complexity of influential social structures in these contexts which is common in health and social care and provides a lens to examine what works through the study of causal mechanisms (Koopmans and Schiller, 2022; Williams et al., 2017). Whilst randomised controlled trials are a highly valued method in healthcare research for attributing causation, realism challenges the lack of external validity in such studies (Emmel et al., 2018; Wallace et al., 2022). Interventions such as blended diets cannot be blinded to increase validity of randomised studies and require observation within their context hence a realist stance is suited to this research.

**Figure 1.**
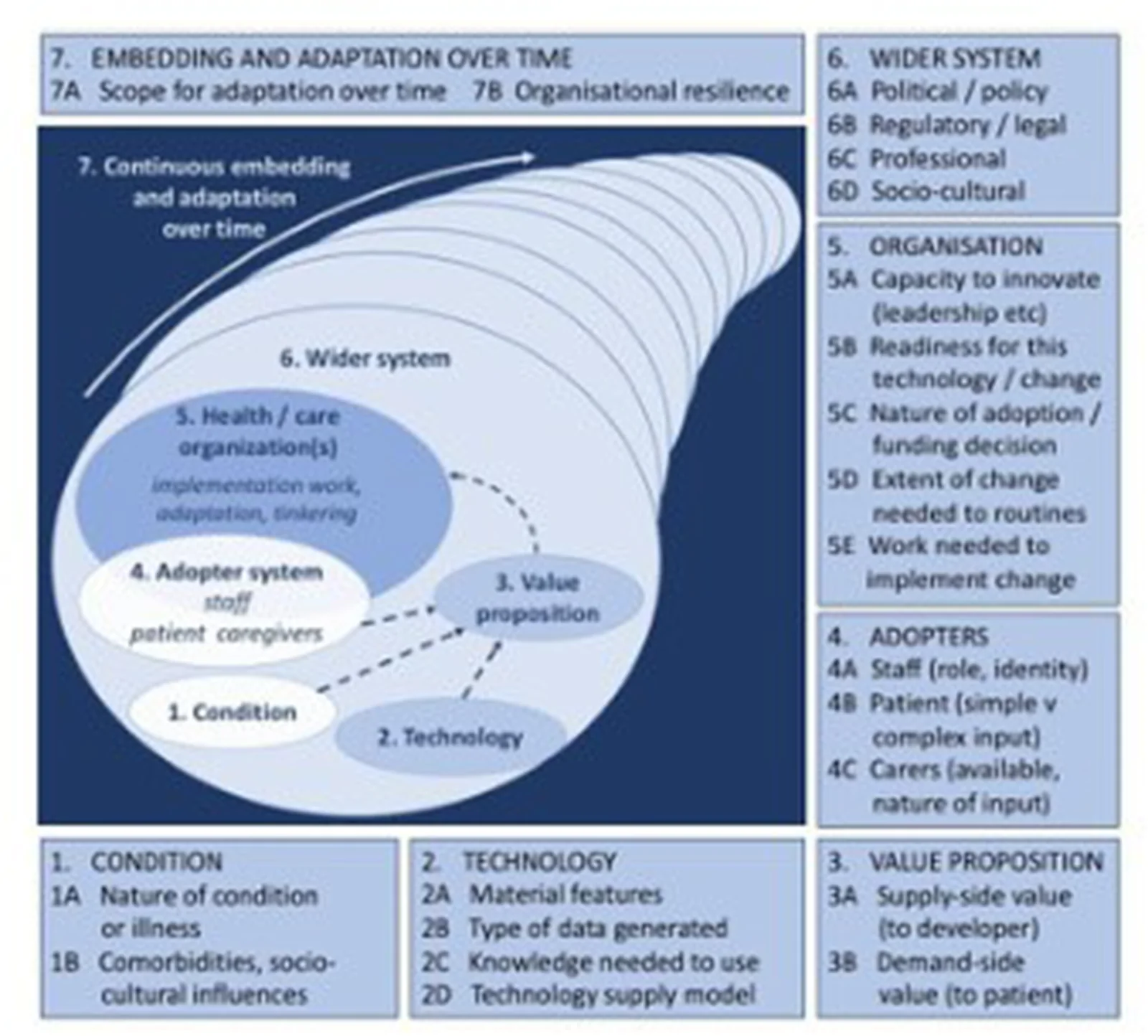
The non-adaption, abandonment, scale-up spread and sustainability (NASSS) framework (Greenhalgh et al., 2017). Source: ©Trisha Greenhalgh, Joseph Wherton, Chrysanthi Papoutsi, Jennifer Lynch, Gemma Hughes, Christine A'Court, Susan Hinder, Nick Fahy, Rob Procter, Sara Shaw. Originally published in the Journal of Medical Internet Research (http://www.jmir.org), 01.11.2017.

The generation of context, mechanism and outcome configurations (CMOCs) is central to realist research to generate and refine a programme theory (Pawson and Tilley, 1997). In this study, initial programme theory will be generated by the researcher and key stakeholders with reference to the evidence-base. The Non-adoption, Abandonment, Scale-up, Spread and Sustainability (NASSS) framework (Greenhalgh et al., 2017) will be used to ensure wider influences are considered. This provides an overarching framework to situate the study and the influences on the use of blended diets (see Figure 1).

### Study design

**Figure 2.**
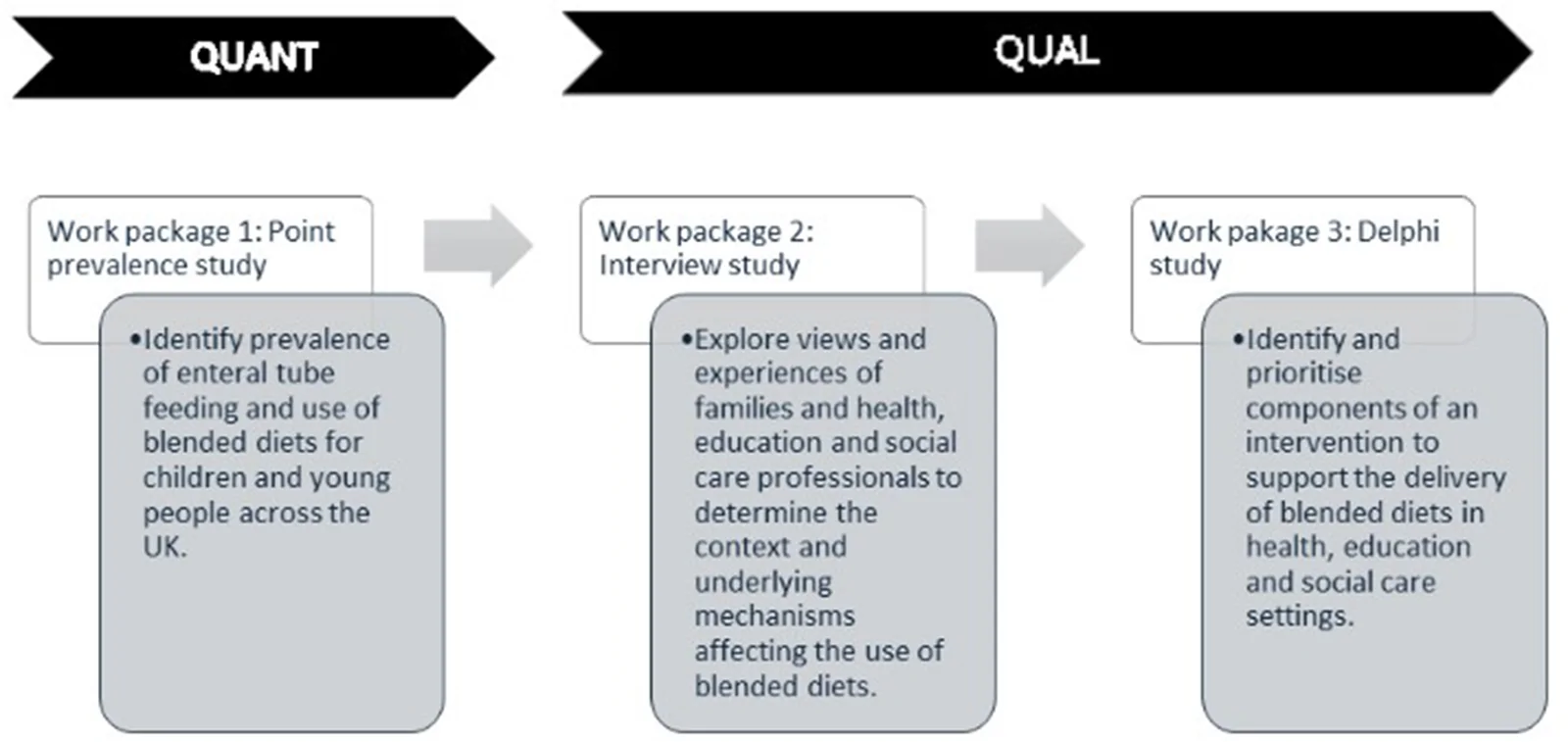
Mixed methods study design.

This study uses a sequential mixed methods design consisting of three work packages to achieve the study aim (see Figure 2). Quantitative data will demonstrate the scale of blended diet use whilst qualitative methods will capture the voice of families and professionals with experience of blended diets to meet the study objectives. This will provide a rich understanding of mechanisms at play and influential contexts. Previous research and PPIE has highlighted that families have significant expertise due to the role of families in raising the profile of blended diets.

Integration is key to mixed methods studies whereby both methods ‘are in dialogue’ (Plano Clark, 2019) with each other. In this study, findings from the point prevalence study (work package 1) will inform the sampling of participants for the interview study (work package 2). Participants for the interview study (work package 2) will be recruited through NHS organisations with high prevalence of blended diet use. This will support rich data collection about the contexts and mechanisms supporting their use. Initial rounds of the Delphi study (work package 3) will be informed by the interview data so that contextual factors and influential mechanisms identified can be ranked and prioritised ahead of subsequent stages of intervention development.

Mixed methods studies are suited to understanding complex phenomena which cannot be adequately explored using qualitative or quantitative methods alone (Plano Clark, 2017). Mixed methods research supports an understanding of ‘how’ and ‘why’ rather than simply ‘what’ which is key in this research which is complex and nuanced. The aim of using mixed methods in this study is to establish generative causation, necessary for realist research; quantitative methods can identify patterns whilst qualitative insights identify the causal mechanisms.

A Good Reporting of a Mixed Methods Study (GRAMMS) checklist can be seen in Appendix 1 (O'Cathain et al., 2008).

### Participants

Families of children and young people using enteral feeding tubes are the focus of this research. The study focuses on the provision of enteral tube feeds which is predominantly a parental responsibility; hence, participants will be adults (parents/carers) or young people (over 16 years) if they manage their own enteral tube feeding. The complexity of these children and young people means that they rely on parents or carers much more than others their age. Consequently, their health and care needs can have a huge impact on the whole family. In this study, the definition of children and young people includes those up to 25 years old in line with government legislation related to special educational needs and disabilities which are common in this group (Department for Education and Health, 2015).

The study will use the following inclusion and exclusion criteria:

#### Work package 1

Inclusion:

- National Health Service provider in the UK

Exclusion:

- Health service providers known to not be involved in home enteral tube feeding (e.g. ambulance trusts)

#### Work packages 2 and 3

Inclusion:

- Parent or main carer of a child or young person (aged 0–25 years) or young person aged 16–25 years using home enteral tube feeding in the UK.
- Professionals who work for an organisation within health, education or social care in the UK providing services to children and young people using home enteral tube feeding.
- Interview study only: Access or work within organisations associated with NHS Trusts identified in point prevalence study.

### Sampling and recruitment

All NHS organisations will be considered for inclusion in the point prevalence study which will identify the number of people using home enteral tube feeding and of those, the proportion using a blended diet. They will be contacted using freedom of information act requests to obtain data on prevalence of home enteral tube feeding and use of blended diets.

Purposive sampling for the interview and Delphi study will ensure that participants have the relevant knowledge, experience and expertise of blended diets necessary to gather rich, in-depth data (Palinkas et al., 2015). Participants will be recruited through NHS trusts and health boards and other organisations such as special needs schools and hospices to achieve a diverse sample. Eligibility criteria for the interview and Delphi study are the same, and participants can partake in one or both. Additional participants may also be recruited using a snowballing method. Authors acknowledge that participants included in the study may have experienced different or fewer barriers to families without awareness or experience of blended diets. However, an understanding of ways that barriers have been overcome was deemed an insightful first step in developing knowledge on the topic.

A sampling framework will be used to capture those with a range of experience (length of time using a blended diet or professional background). The framework provides an approximate sample size and may be adjusted according to the conceptual depth of the data collected (see Appendix 2). Multi-disciplinary Delphi panels need to be larger to capture a range of participants as is the case with this study (Manyara et al., 2024). In addition, PPIE representatives have explained that due to the fluctuating care needs of their children, their availability to partake may vary. For this reason, a larger panel may be necessary to accommodate a higher than usual drop-out rate. It is generally advised to seek a minimum panel size and ensure representation of subgroups (Beiderbeck et al., 2021; Page et al., 2015).

Participants will contact the researcher if they would like to take part in the study. They will be sent a participant information sheet and an expression of interest form to complete. This will include demographic information, contact details and criteria required to assess suitability based on the sampling framework in addition to any communication needs that need to be accommodated by the researcher. Eligible participants will then be contacted by the researcher to agree participation and complete the consent process.

### Data collection

#### Work package 1

Quantitative data collected in the point prevalence study will be obtained using freedom of information act requests. Returned data will be cleaned and standardised ahead of analysis. The information request will be piloted on 5% of sites initially and any changes made as required.

#### Work package 2

Interviews will be semi-structured to allow flexibility whilst maintaining structure to cover key concepts. The interview guide will be developed by the researcher and reviewed by academics, stakeholders and patients and members of the public. This includes the research team consisting of nurses and dietitians and wider professional stakeholder group (including paediatricians and speech and language therapists) who will meet online to discuss the guide. PPIE input will be provided by a group of parent carers involved in the study throughout who will be involved to pilot the interview guide. Interview questions will be informed by the theoretical domains framework to elicit understanding of behavioural influences (Atkins et al., 2017). Interviews will be carried out remotely using videoconferencing software or by phone as per participant preference. Face-to-face interviews may be accommodated if preferred. Interviews will be recorded and transcribed verbatim ahead of data analysis. Single interviews have been chosen to minimise burden on participants who are likely to have significant health needs or caring responsibilities. Interviews will be scheduled at times to suit the participant.

#### Work package 3

The Delphi study will consist of up to three rounds. The first will be based on findings from the interview study including themes generated from the analysis. These will highlight identified barriers and strategies to address them which participants will assess and determine their importance. Free text boxes will allow participants to suggest ways that these barriers may be addressed. Rounds two and three will gain consensus on barriers and prioritise ways that they can be addressed which will inform the development of an intervention. A cut off between 70% and –80% will be used and agreed with the team with input from stakeholders and patients and members of the public. Given that this research is exploratory and comprises early stages of complex intervention development, a lower level of agreement may be acceptable.

### Data analysis

The point prevalence of blended diet use will be calculated for each organisation and descriptive statistics (e.g. mean, median) will be calculated for the whole data set as well as separately for children (0–17 years) and young people (18–25 years).

Reflexive thematic analysis will be used for the interview study (Braun and Clarke, 2019). This supports a realist approach to offering an explanatory understanding of the context and mechanisms affecting the use of blended diets recognising the co-creation of knowledge between the researcher and the data. A proportion of the interview recordings will be transcribed by the researcher to aid familiarisation; any transcribed externally will be read multiple times. Interview transcripts will be coded by the researcher by identifying meaningful words and phrases. Codes will then be grouped into themes and labelled to summarise important topics in the data collected.

Coded data will also be used to refine the programme theory. Data will provide evidence to confirm, refine or refute the programme theory through an iterative process involving stakeholders and patients and members of the public. Causal mechanisms and their influence on outcomes of interest will be explained using CMOCs.

Data analysis in the Delphi study will involve calculating frequencies of responses, medians and interquartile range of Likert scales. Qualitative data from free text responses will be analysed using reflexive thematic analysis as with the interview data. Response rates, drop-outs and characteristics of the panel will be monitored and reported in the study findings. Data from the Delphi study will be used to further refine the programme theory through iterations of the CMOCs.

### Reflexivity

Researcher reflexivity is integral due to the qualitative nature of the study. Reflexive journaling and team discussions will capture how researcher beliefs and experiences influence the study (Olmos-Vega et al., 2023). These influences will be reported with the findings of the study to highlight the impact on decision making throughout the study.

### Equality impact assessment

Equality impact assessments are becoming increasingly embedded in health and social care research to understand how research practices affect people differently and ways that they can be accommodated (NIHR, 2025). Minority groups have historically been excluded from research and it is necessary that they are included to ensure that clinical practice is underpinned by inclusive research to be truly evidence-based (Brooker et al., 2014). Given the focus of enteral tube feeding, it was deemed likely to positively impact people with disabilities by advancing knowledge on a topic relevant to this under-served group. The assessment involved consideration of all protected characteristics to optimise inclusion in the study. Some methodological considerations resulting from the equality impact assessment can be seen in Box 1.

Box 1. Methodological considerations resulting from equality impact assessment

Age:

- The age range of children and young people is inclusive of young people up to 25 years.
- The readability of participant information materials has been assessed to ensure that they are suitable for the target population.

Disability:

- Remote data collection methods, for example, online or phone interviews were deemed more suitable for this group by patients and members of the public.

Race or religion:

- Recruitment methods include sampling through the NHS to increase opportunity for participants to be involved.
- Timing of data collection will aim to avoid religious festivals such as Ramadan.
- Flexibility when planning interviews will accommodate people from different groups such as those who work or have caring responsibilities.
- The research has been designed with input from a diverse PPIE group.

### Patient and public involvement and engagement

**Figure 3.**
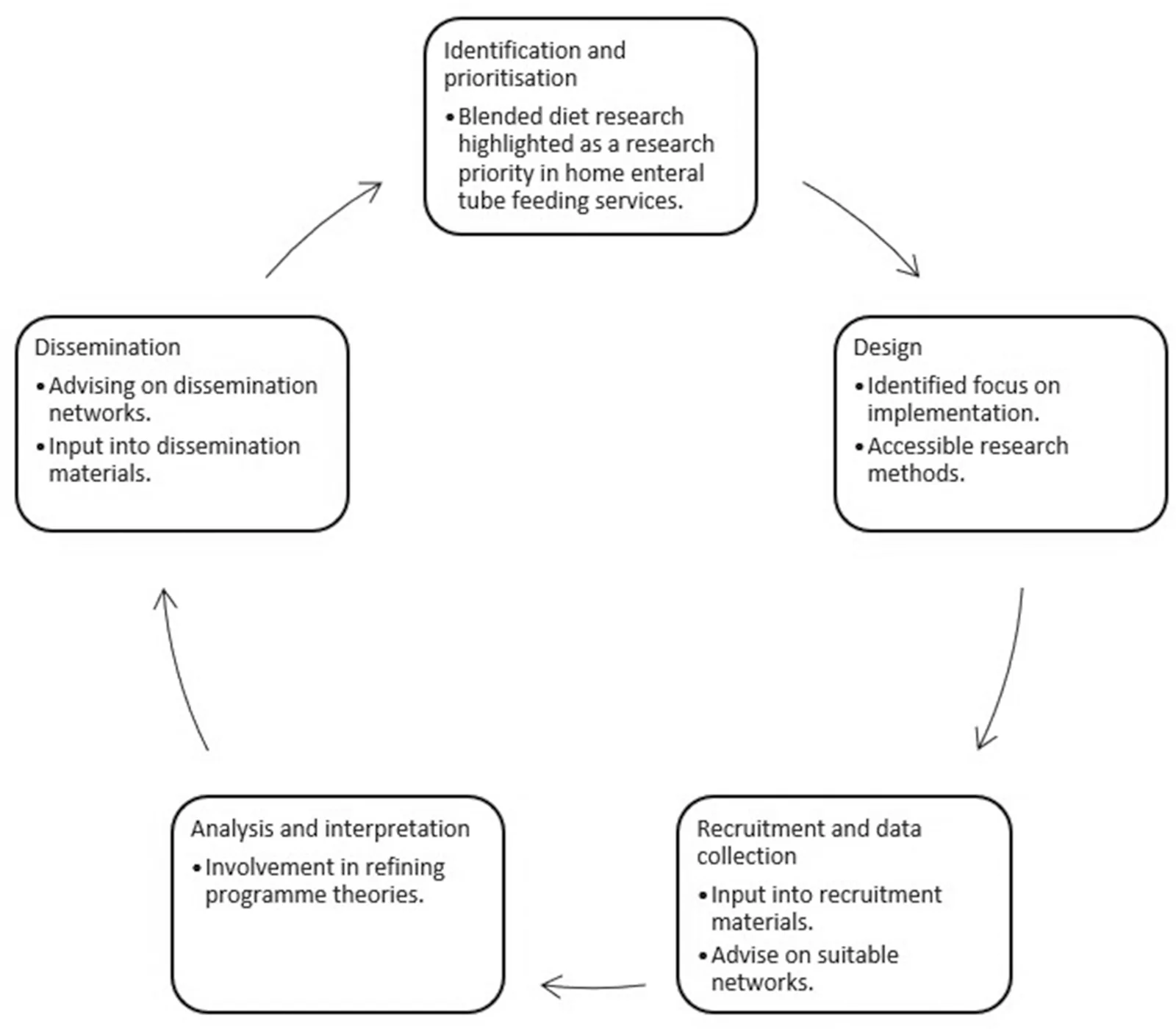
Patient and public involvement and engagement (PPIE) input.

Inclusion of PPIE in healthcare research adds invaluable insight which can optimise the quality, relevance and application of the research (Arumugam et al., 2023). Figure 3 shows some examples of how PPIE has been and will be embedded in this study.

Early PPIE identified the need for research on this topic. Formal PPIE activity informed the study design and grant application for this research and identified that subsequent PPIE must accommodate those with significant caring responsibilities of children and young people with fluctuating health. As a result, a flexible approach using remote ways of working has been incorporated in this study.

The group consists of parent carers of tube-fed children and young people, both male and female from a range of ethnic backgrounds. Group members were invited through a national tube feeding charity (PINNT), social media and clinicians involved in the study. Group members are invited to meetings or asked for input, with the option to participate as their availability allows, without pressure to commit. Online meetings have proven effective; those unable to attend can stay informed through meeting minutes or recordings. PPIE input has shaped recruitment materials, imagery and the wording of participant information sheets. Their involvement in reviewing aspects such as survey length has helped ensure that participation is not burdensome, thereby enhancing accessibility.

## Ethics and dissemination

Ethical approval for this study has been granted by NHS Health Research Authority South Birmingham Research Ethics Committee (REC reference: 25/WM/0099).

All participants will provide informed consent and may withdraw at any time without giving a reason. Participant information sheets (PIS) have been assessed for readability and written in plain English. All data will be stored securely, and interview transcripts will be pseudonymised on transcription.

Young people (16–25 years) are eligible to take part and a separate PIS will be developed including an easy read version for inclusivity. Young people can choose to bring someone with them if they choose. The researcher will complete interviews in a private room to maintain confidentiality of participants. The interview topic is not inherently distressing; however, interview will be paused if the participant becomes distressed. In this instance, participants will be offered the choice to continue, postpone or terminate the interview.

Participants will be asked to provide an email address if they wish to be updated with the study findings. The research team will write participant summaries in plain English and ask PPIE representatives to review them and advise of other networks to share the study findings. On completion, the study will be submitted for publication in international peer-reviewed journals, presented at topic specific conferences and shared within professional networks. A policy briefing will also support the translation of the findings into practice.

## Summary

This paper describes the protocol for a novel, mixed methods study exploring contexts and mechanisms affecting the use of blended diets for children and young people in health, education and social care settings in the UK. Inspired by families with lived experience of using blended diets, it will progress knowledge of this topic area further expanding the evidence base underpinning practice in this area which is currently lacking. This study will underpin subsequent development of a complex intervention to support the use of blended diets in health, education and social care settings by identifying influential contexts and mechanisms. This understanding will enable subsequent interventions, focusing on the implementation of blended diets, to be appropriately targeted optimising efficiency and effectiveness. The significance of this study relates to the specific requirement identified by families using blended diet. This is the need for research to support the use of blended diets which this study aims to address. Families explained that challenges remain with using blended diets away from home and a lack of consistent support across settings accessed by tube-fed children and young people.

The strengths of this study lie in the use of mixed methods to gain a comprehensive answer to the research question. Data integration is cohesive across the work packages for a truly mixed methods approach. The study is both data-driven and theoretically informed, ensuring that findings will be systematically interpreted and grounded within relevant conceptual frameworks. A realist lens ensures that the phenomena are studied within context enhancing external validity and therefore applicability to policy and practice. A nationwide approach will increase the transferability of findings and inform a standardised approach to development in this area of practice for those accessing NHS services.

Findings from this study will inform future research on interventions to better support blended diets to optimise choice. It provides a rigorous foundation for future developments in this area centred around the needs of children, young people and their families.

## Supplemental Material

sj-docx-1-nah-10.1177_02601060261439096 - Supplemental material for Exploring factors affecting the use of blended diets for children and young people in health, education and social care settings in the UK: The protocol for a realist mixed methods study Supplemental material, sj-docx-1-nah-10.1177_02601060261439096 for Exploring factors affecting the use of blended diets for children and young people in health, education and social care settings in the UK: The protocol for a realist mixed methods study by Gemma Phillips, Jane Coad, Mary Hickson and Joseph C Manning in Nutrition and Health

sj-docx-2-nah-10.1177_02601060261439096 - Supplemental material for Exploring factors affecting the use of blended diets for children and young people in health, education and social care settings in the UK: The protocol for a realist mixed methods study Supplemental material, sj-docx-2-nah-10.1177_02601060261439096 for Exploring factors affecting the use of blended diets for children and young people in health, education and social care settings in the UK: The protocol for a realist mixed methods study by Gemma Phillips, Jane Coad, Mary Hickson and Joseph C Manning in Nutrition and Health
