# Supplementary material for: Exploring factors affecting the use of blended diets for children and young people in health, education and social care settings in the UK: The protocol for a realist mixed methods study
Source: Nutr Health. 2026 Apr 8;32(5):1419–28. doi: 10.1177/02601060261439096 (PMC13538907; doi:10.1177/02601060261439096)
Supplement: Supplementary file 1 [file sj-docx-1-nah-10.1177_02601060261439096.docx]

Appendix 1

Good reporting of a mixed methods study (GRAMMS) checklist

| Guideline | Section: page |
| --- | --- |
| Describe the justification for using a mixed methods approach to the research question | Study design p. 6 |
| Describe the design in terms of the purpose, priority and sequence of methods | Study design p. 6 |
| Describe each method in terms of sampling, data collection and analysis | Data collection p. 6-8 |
| Describe where integration has occurred, how it has occurred and who has participated in it | Study design p. 6 |
| Describe any limitation of one method associated with the present of the other method | NA for protocol paper |
| Describe any insights gained from mixing or integrating methods | Study design p. 6 |

(O’Cathain et al., 2008)
