# Supplementary material for: Exploring factors affecting the use of blended diets for children and young people in health, education and social care settings in the UK: The protocol for a realist mixed methods study
Source: Nutr Health. 2026 Apr 8;32(5):1419–28. doi: 10.1177/02601060261439096 (PMC13538907; doi:10.1177/02601060261439096)
Supplement: Supplementary file 2 [file sj-docx-2-nah-10.1177_02601060261439096.docx]

Appendix 2

Sampling framework for qualitative studies

|  | **Parents, main carers and young people** | **Professionals** |  |
| --- | --- | --- | --- |
| **Interview study** | Up to 20 participants with diversity across the following groups:   - <2 years using a blended diet - 2-5 years using a blended diet - Over 5 years using a blended diet | Up to 20 with variation across professional groups and setting where they are based e.g. acute, community, health, education or social care. |  |
| **Delphi study** | Minimum of 15 participants with diversity across the following groups:   - <2 years using a blended diet - 2-5 years using a blended diet - Over 5 years using a blended diet | Minimum of 15 participants with variation across professional groups and setting where they are based e.g. acute, community, health, education or social care. |  |
|  |  |  |  |
